# Identification of Key Features Pivotal to the Characteristics and Functions of Gut Bacteria Taxa through Machine Learning Methods

**DOI:** 10.2174/0115665232367064250630202337

**Published:** 2025-07-15

**Authors:** ZhanDong Li, QingLan Ma, Hao Li, Lin Lu, Lei Chen, Wei Guo, KaiYan Feng, Tao Huang, Yu-Dong Cai

**Affiliations:** 1 School of Biological and Food Engineering, Jilin Engineering Normal University, Changchun 130052, China;; 2 School of Life Sciences, Shanghai University, Shanghai 200444, China;; 3 Department of Radiology, Columbia University Medical Center, New York 10032, USA;; 4 College of Information Engineering, Shanghai Maritime University, Shanghai 201306, China;; 5 Key Laboratory of Stem Cell Biology, Shanghai Jiao Tong University School of Medicine (SJTUSM) & Shanghai Institutes for Biological Sciences (SIBS), Chinese Academy of Sciences (CAS), Shanghai 200030, China;; 6 Department of Computer Science, Guangdong AIB Polytechnic College, Guangzhou 510507, China;; 7 Bio-Med Big Data Center, CAS Key Laboratory of Computational Biology, Shanghai Institute of Nutrition and Health, University of Chinese Academy of Sciences, Chinese Academy of Sciences, Shanghai 200031, China;; 8 CAS Key Laboratory of Tissue Microenvironment and Tumor, Shanghai Institute of Nutrition and Health, University of Chinese Academy of Sciences, Chinese Academy of Sciences, Shanghai 200031, China

**Keywords:** Gut bacteria, machine learning, feature selection, disease pathology, ecosystem, gut dysbiosis

## Abstract

**Background:**

Gut bacteria critically influence digestion, facilitate the breakdown of complex food substances, aid in essential nutrient synthesis, and contribute to immune system balance. However, current knowledge regarding intestinal bacteria remains insufficient.

**Objective:**

This study aims to discover essential differences for different intestinal bacteria.

**Methods:**

This study was conducted by investigating a total of 1478 gut bacterial samples comprising 235 *Actinobacteria* , 447 *Bacteroidetes* , and 796 *Firmicutes* , by utilizing sophisticated machine learning algorithms. By building on the dataset provided by Chen *et al.* , we engaged sophisticated machine learning techniques to further investigate and analyze the gut bacterial samples. Each sample in the dataset was described by 993 unique features associated with gut bacteria, including 342 features annotated by the Antibiotic Resistance Genes Database, Comprehensive Antibiotic Research Database, Kyoto Encyclopedia of Genes and Genomes, and Virulence Factors of Pathogenic Bacteria. We employed incremental feature selection methods within a computational framework to identify the optimal features for classification.

**Results:**

Eleven feature ranking algorithms selected several key features as pivotal to the characteristics and functions of gut bacteria. These features appear to facilitate the identification of specific gut bacterial species. Additionally, we established quantitative rules for identifying *Actinobacteria* , *Bacteroidetes* , and *Firmicutes* .

**Conclusion:**

This research underscores the significant potential of machine learning in studying gut microbes and enhances our understanding of the multifaceted roles of gut bacteria.

## 1 INTRODUCTION

 The human gut microbiota represents a diverse ecosystem teeming with trillions of bacteria, viruses, and other microbes, which are predominantly from the *Firmicutes* and *Bacteroidetes phyla* [1, 2]. This ecosystem is pivotal in sustaining human health and modulating disease pathology [3, 4]. Its primary constituents, the intestinal bacteria, offer substantial benefits through energy extraction, vitamin synthesis, immune system regulation, and pathogen competition [5-8]. Nevertheless, imbalances in these microbial communities, termed "gut dysbiosis," can trigger a spectrum of health complications that range from localized inflammatory conditions to systemic diseases, such as obesity, diabetes, and even neurodegenerative disorders [9-12]. Numerous factors, including genetics, diet, lifestyle, and medication usage, influence the unique composition of an individual's gut microbiota, thereby shaping disease susceptibility and progression [13-16]. Consequently, a comprehensive understanding of key bacterial groups, including *Actinobacteria*, *Bacteroidetes*, and *Firmicutes*, is essential. Profiling these groups and elucidating their roles within the gut ecosystem may provide valuable insights into disease mechanisms and inform potential therapeutic strategies.

Three essential constituents of the human gut microbiota—*Actinobacteria*, *Bacteroidetes*, and *Firmicutes*—each perform unique, essential roles within the intestinal ecosystem. Specifically, *Actinobacteria*, particularly the Bifidobacteria family, are central to gut homeostasis, regulating gut permeability, immune response, metabolism, and the gut–brain axis [17-19]. *Bacteroidetes* are key to the fermentation of complex polysaccharides and host defense against pathogens, and play a role in influencing metabolism, host physiology, gut motility, and barrier integrity [20-27]. *Firmicutes*, meanwhile, assist in deriving energy from complex sugars and proteins and contribute to the generation of short-chain fatty acids, a vital energy source for colon cells [25, 28]. In addition, the *Firmicutes*:*Bacteroidetes* ratio in the gut has been studied in relation to obesity [29]. Nonetheless, further research remains necessary to unveil the crucial roles played by these three types of gut microbiota and develop appropriate techniques for their identification.

Despite the advances in sequencing technologies, understanding the complex structure and function of the gut microbiome remains a challenge. Functional annotation–based approaches have emerged as a promising alternative for characterizing the gut microbiome, offering several advantages over traditional sequencing methods. These approaches include the highly targeted and efficient analysis of large-scale datasets [30], a highly detailed and comprehensive understanding of functional capacity [31], and improved accuracy and robustness of classification and prediction models [32]. In light of these advantages, our study aims to apply a functional annotation–based approach to identify the key functional signatures that reflect differences in functional levels and explore their potential applications in understanding the human gut microbiome.

In our study, we strove to understand the functional differences and distributions of bacterial subgroups and devised efficient classification rules. We reanalyzed data from Chen *et al.* [33], who utilized only one feature sorting algorithm, Monte Carlo feature selection (MCFS), to analyze the data, which may be incomplete. We used an innovative approach that employed 11 unique feature ranking algorithms, studying a diverse sample of 1478 bacterial strains. Chen *et al.* analyzed the functional characteristics of intestinal bacteria by applying a single MCFS method and initially revealed the differences in the characteristics of different bacterial phyla. On the basis of this study, 11 different feature ranking algorithms were used to analyze the functional characteristics of intestinal bacteria comprehensively and systematically to reveal the differences in the characteristics of different bacterial phyla from multiple perspectives. In addition, we screened the optimal feature combinations through incremental feature selection (IFS) [34] and constructed classification rules to reflect differences between the three types of phyla at functional levels. Our data were dissected into a total of 993 distinct features, which were categorized by the Antibiotic Resistance Genes Database (ARDB) [35], Comprehensive Antibiotic Research Database (CARD) [36], Kyoto Encyclopedia of Genes and Genomes (KEGG) [37], and Virulence Factors of Pathogenic Bacteria (VFDB) [38]. By implementing multiple feature sorting algorithms, we constructed an importance-ordered list of features. Furthermore, we employed the IFS method to curate an optimal feature subset for accurate bacterial classification. By using decision trees (DTs), we established quantitative rules for identifying *Actinobacteria*, *Bacteroidetes*, and *Firmicutes*. Importantly, our study managed to identify certain critical biomarkers associated with the human gut microbiota. This discovery serves as an important milestone in gaining a deepened and nuanced understanding of the gut microbiota, with potential implications for health and disease understanding.

## 2 MATERIALS AND METHODS

### 2.1 Data

In this study, we used the data provided by Chen *et al.* [33] as our primary source of data. It is a systematic dataset on the gut microbiome from approximately 1500 human gut bacterial strains downloaded from the China National GeneBank under Project ID: CNP0000126. The dataset comprises 1478 distinct intestinal bacteria, specifically consisting of 235 *Actinobacteria*, 447 *Bacteroidetes*, and 796 *Firmicutes*. Each sample in the dataset encompasses 993 informative features, including 342, 259, 243, and 149 features annotated by ARDB, CARD, KEGG, and VFDB, respectively. ARDB features describe the functional characteristics related to antibiotic resistance by microbial gene annotation. CARD features summarize resistance determinants and predict resistance genes on the basis of resistance ontology and detection models. VFDB features annotate virulence factors and potential pathogens. KEGG features characterize the functional roles of genes [35-40]. Collectively, these four feature sets annotate bacterial genes to generate a mix of qualitative and quantitative features describing antibiotic resistance, virulence, metabolic pathways, and gene functions.

### 2.2 Feature Ranking Methods

Although the analyzed sample exhibited an abundance of features, only a minuscule proportion was associated with the functionalities and properties of particular gut bacteria. We applied a set of 11 disparate feature ranking algorithms to enhance our comprehension of these gut bacteria. These algorithms included Adaptive Boosting (AdaBoost) [41], Categorical Boosting (CatBoost) [41], Extremely Randomized Trees (ExtraTrees) [42], Least Absolute Shrinkage and Selection Operator (Lasso) [43], Light Gradient Boosting Machine (LightGBM) [44], MCFS [45], Minimum Redundancy Maximum Relevance (mRMR) [46], Random Forest (RF_ZL) [47], Ridge Regression (Ridge) [48], Support Vector Machine-Recursive Feature Elimination (SVM-order) [49], and eXtreme Gradient Boosting (XGBoost) [50]. These algorithms can overview the investigated dataset from different aspects, thereby nearly fully evaluating the importance of features.

#### 2.2.1 AdaBoost

AdaBoost is one of the most popular and influential ensemble methods in machine learning [51]. Introduced by Freund and Schapire in 1996, it is an algorithm that combines several weak learners into a strong learner to improve prediction accuracy. It functions by creating a sequence of models that attempt to correct the mistakes of the preceding models in a sequence. Feature importance in AdaBoost is computed on the basis of the total amount of weights that each feature contributes to each decision stump (one-level DT) across all of the iterations of boosting. The weighted error rate of a decision stump is a measure of how well it classifies training instances. In this context, the importance of a feature is proportional to the weights of the decision stumps using that feature. We downloaded Adaboost from https://scikit-learn.org/stable/modules/generated/sklearn.ensemble.AdaBoostClassifier.html#sklearn.ensemble.AdaBoostClassifier and used default parameters.

#### 2.2.2 CatBoost

The CatBoost is a gradient boosting library developed by Yandex that stands out for its ability to handle categorical variables effectively [41]. The name CatBoost is derived from the words "category" and "boosting". While traditional gradient boosting algorithms require categorical variables to be preprocessed, CatBoost handles categorical variables internally, saving time and potentially improving model performance. The feature importance provided by CatBoost is prediction value change, which refers to the alteration in the predicted outcome or result when certain variables are modified or permutated. It is a measure of how sensitive a prediction is to changes in input variables. The prediction value change can provide insights into the importance and influence of specific features on the overall prediction. By analyzing the magnitude of the prediction value change, we can understand the degree to which modifications in input variables affect the final prediction and use it to estimate feature importance. In this study, CatBoost was acquired from https://catboost.ai/en/docs/features/feature-importances-calculation, and default parameters were utilized for the analysis.

#### 2.2.3 ExtraTrees

ExtraTrees is an ensemble learning method that is fundamentally based on DTs. It shares numerous similarities with the well-known random forest (RF) algorithm, but with a crucial difference in the way the nodes of trees are split [42]. ExtraTrees selects random thresholds for each feature rather than searching for the best possible thresholds like a DT would. Once all trees have been fitted, the feature importance for a single feature is computed as the average across all the trees of the impurity decrease for that feature, normalized by the total impurity decrease for all features. Compared with an RF algorithm, the ExtraTrees algorithm introduces additional randomness into the construction of the trees. This approach can increase diversity among the trees and in turn lead to feature importance scores with increased stability and reliability. We obtained ExtraTrees from https://scikit-learn.org/stable/modules/generated/sklearn.ensemble.ExtraTreesClassifier.html#sklearn.ensemble.ExtraTreesClassifier.feature_importances_ and utilized default parameters for analysis.

#### 2.2.4 Lasso

Lasso is a regression analysis method that performs variable selection and regularization to enhance the prediction accuracy and interpretability of the statistical model it produces [43]. Although Lasso was originally formulated for linear regression models, it can be used with various types of regression models as well. In terms of feature importance, Lasso uses L1 regularization, which adds a penalty equivalent to the absolute value of the magnitude of the coefficients. This L1 penalty has the effect of forcing some of the coefficient estimates to be exactly equal to zero when the regularization strength lambda is sufficiently large. This effect leads to a sparse model, wherein only a subset of the predictors is left with nonzero coefficients. These nonzero coefficients are therefore the selected features, and their magnitude gives an indication of their importance in the model. Large absolute values of the Lasso coefficient for a feature indicate the high importance of that feature. Here, the Lasso program was sourced from scikit-learn [52]. It was run with default parameters and alpha set at 0.1 as the main parameter for convenience.

#### 2.2.5 LightGBM

LightGBM is a gradient boosting framework developed by Microsoft that uses tree-based learning algorithms [44]. It is designed to be efficient and accelerate training and handling large-scale data without sacrificing accuracy. Feature importance provided by the LightGBM is estimated by gain importance. It computes the total gain of each feature when it is used in trees. In other words, it measures the total improvement in the model as a result of splits over a particular feature. A feature with a higher value of this metric than another feature implies that it is more important for generating a prediction. Gain importance helps understand which features are the driving factors behind the decision made by the LightGBM model, and these features can help guide feature selection in future modeling. In this research, the LightGBM program was acquired from the official website at https://lightgbm.readthedocs.io/en/latest/. Analysis was conducted by utilizing default parameters (refer to https://lightgbm.readthedocs.io/en/latest/Parameters.html#core-parameters for detailed information).

#### 2.2.6 MCFS

The MCFS method developed by Draminski *et al.* is a robust technique for assessing the importance of features, especially in high-dimensional datasets [45]. The main idea of MCFS is to construct a large number of DTs on randomly selected subspaces of the dataset (hence the name "Monte Carlo") and then ranking the features in accordance with their importance. A subset of instances and another of features are randomly selected from the original dataset. This random selection introduces the "Monte Carlo" aspect into the procedure. A DT is built on the selected data subspace. This tree is pruned to a certain size to avoid overfitting. After building the DTs, each feature is assigned a score estimating its importance on the basis of the weighted accuracy of each DT, information gain, and its coverage in each node. Features can then be sorted by importance scores. In this study, the MCFS program was utilized. The program can be obtained from http://www.ipipan.eu/staff/m.draminski/mcfs.html or https://github.com/chenlei1982/CHD_SingleCell/tree/main/Feature-ranking-algorithms/MCFS and executed with default parameters. The primary parameters *u* and *v*, used for calculating the RI, were set to 1.

#### 2.2.7 mRMR

mRMR is a feature selection method used in machine learning and statistics, making the features that ranked highest possess the highest mutual information with the target variable while also ensuring that they are as mutually dissimilar as much as possible [46]. The concept of mutual information here is based on the concept of entropy in information theory. mRMR aims to balance two criteria: maximum relevance and minimum redundancy. The principle of maximum relevance focuses on the individual predictive capability of each feature. After maximum relevance is considered, mRMR examines the redundancy among the selected features. Its aim is to minimize the mutual information between features, *i.e*., ensuring that each new feature adds new information to the model and is not just repeating the information of already selected features. In this research, the mRMR program from http://home.penglab.com/proj/mRMR/ was utilized. The program was executed with default parameters, and the selection scheme used was mutual information difference.

#### 2.2.8 RF

RF, an ensemble machine learning method, provides a straightforward and effective way to measure feature importance, making it a valuable tool not only for prediction but also for understanding the underlying structure of the data [47]. It estimates the importance of a feature by calculating the mean decrease impurity from the decrease in node impurity weighted by the probability of reaching that node (which is approximated by the proportion of samples reaching that node). A high value is indicative of a highly important feature. RF's method of assessing feature importance is particularly valuable because it takes into account interaction effects between features. It does not require any additional steps, such as training a separate model, thus making it efficient. We downloaded RF_ZL from the https://scikit-learn.org/stable/modules/generated/sklearn.ensemble.RandomForestClassifier.html#sklearn.ensemble.RandomForest Classifier.feature_importances_ and used the default parameters.

#### 2.2.9 Ridge Regression

Ridge Regression, also known as Tikhonov regularization, is a technique used in linear regression to prevent overfitting [48]. Ridge Regression introduces a regularization term (a penalty) to the loss function of the linear regression model. The regularization term is the squared L2 norm of the feature weights (hence Ridge) multiplied by a tuning parameter, lambda (also known as alpha). In a Ridge Regression model, the importance of a feature is generally proportional to the absolute value of its corresponding coefficient. Features with large absolute value coefficients are considered important because a change in these features has a large effect on the predicted outcome. If the scales of the features differ, a small coefficient associated with a feature that has a large scale can actually be more important than a large coefficient associated with a feature that has a small scale. For a fair comparison of the coefficients, features must be ensured to have been scaled to have a similar range, for example, by using standardization. In this research, the Ridge classifier was obtained from the scikit-learn library (https://scikit-learn.org/stable/modules/generated/sklearn.linear_model.RidgeClassifier.html#sklearn.linear_model.RidgeClassifier), and its default parameters were employed for analysis.

#### 2.2.10 SVM-RFE

SVM-RFE is a type of feature selection technique based on the support vector machine (SVM) algorithm [49]. The SVM-RFE method ranks features on the basis of their ability to separate the classes under consideration. A general description of how SVM-RFE works is provided as follows: 1. The SVM model is trained on the dataset. 2. The weight coefficients for each feature are calculated. In SVM, these weights represent the contribution of each feature to the decision boundary. Features with high absolute weight values contribute significantly to the model's output. 3. The feature with the smallest absolute weight value is identified because it is likely to be the least important among all features. 4. The identified feature is removed from the dataset. 5. Steps 1–4 are repeated until all features have been ranked. The concept behind SVM-RFE is that by repeatedly eliminating the feature with the least importance (smallest absolute weight), we can incrementally remove the features with the smallest contribution to the decision boundary, thereby ranking the remaining features by their importance. In this investigation, we acquired SVM-order from the repository available at https://github.com/chunyanyin11/SVMraninking/blob/master/code%20SVM_copd.ipynb and utilized its default parameters for analysis.

#### 2.2.11 XGBoost

XGBoost is an optimized distributed gradient boosting library designed to be highly efficient, flexible, and portable [50-52]. Developed by Chen, it aims to provide a "push-button" solution to high-performing machine learning models. XGBoost provides a measure of how "important" each feature is in contributing to the model's predictions. Feature importance is estimated by the total gain of splits that use the feature. The gain of each feature used in each split of the model is tracked. Subsequently, for each feature, these gains are added together to represent its importance. The idea here is that if splits on a particular feature result in a large improvement in the model's performance, that feature is likely to be important. However, the feature importance in the XGBoost may be biased toward continuous features or high-cardinality categorical features because such features can have more unique split-points than other features and thus may be used in splits more often. In this study, XGBoost was obtained from the official documentation at https://xgboost.readthedocs.io/en/stable/python/python_api.html#xgboost.XGBClassifier.feature_importances_ , and its default parameters were utilized for analysis.

### 2.3 IFS

IFS is a method used in machine learning for selecting the most important features from a list of features [34]. This method is commonly used when dealing with high-dimensional data with numerous features and when reducing dimensionality is desired. The main concept behind IFS is to add one feature at a time on the basis of a certain criterion and then measure the performance of the model with the added feature. Starting from an empty feature set, the process is repeated until all features are added. Finally, a plot with the number of features on the x-axis and the performance metric on the y-axis is created. This plot is known as an IFS curve. The peak of this curve can give an indication of the optimal number of features to retain. The major benefit of IFS is that it provides a clear visualization of how each additional feature affects the performance of the model, allowing for an improved understanding of feature importance and helping in selecting the optimal feature subset.

### 2.4 SMOTE

SMOTE is a popular algorithm used to tackle the issue of imbalance in machine learning datasets wherein the number of instances of one class is considerably lower than that of other(s) [53]. Class imbalance is a common problem in many real-world classification tasks, such as fraud detection, medical diagnosis, and text classification. The standard way of addressing imbalance is by oversampling the minority class, undersampling the majority class, or a combination of both. SMOTE is an oversampling method that generates synthetic samples from the minority class instead of creating copies. A simplified step-by-step procedure is as follows: 1. The distance of one instance in the minority class to other samples in the same class is calculated, and its k-nearest neighbors are selected. 2. A random number between 0 and 1 is selected and used to generate a linear combination of the instance and one of its nearest neighbors. This approach creates a new, synthetic instance somewhere along the line connecting the two instances. 3. The process is repeated until the desired class balance is achieved. In this study, SMOTE was implemented by using the program downloaded from https://github.com/scikit-learn-contrib/imbalanced-learn. The default parameters of the program were adopted for analysis.

### 2.5 Classification Algorithm

Two robust supervised classification algorithms were chosen as the groundwork for implementing the IFS method, specifically, DT [54] and RF [47].

#### 2.5.1 DT

DT is a popular and powerful supervised learning algorithm used for regression and classification tasks [54]. Its name originates from its tree-like structure, wherein each node represents a feature (or attribute), each branch represents a decision rule, and each leaf node represents an outcome. The topmost node in a tree is known as the root node, which contains all instances. The algorithm selects the best attribute to split a node by using a certain metric. The attribute with the "best" split is typically chosen. Splitting stops once a certain condition is met. This situation could occur when all instances in a subset belong to the same class (for a classification problem), when a maximum tree depth is reached, or when splitting a node does not result in sufficient information gain or decrease in impurity. The final result is a tree wherein each branch represents a possible decision path and each leaf node represents a classification or decision. In this implementation of DT, the scikit-learn package was utilized, with Gini coefficients serving as the information gain criterion [52].

#### 2.5.2 RF

RF is a powerful machine learning algorithm that leverages the concept of ensemble learning [47]. It operates by building numerous DTs and then outputting the class that is the mode of the classes for classification problems. It creates multiple subsets from the original dataset. Each subset is used to train a separate DT. The randomness in selecting these subsets ensures that each DT is trained on different data points, adding diversity to the model. At each node in the DT, only a random subset of features is considered for splitting. This random subset of features is different for every node and ensures further randomness in the model, preventing overfitting. Therefore, RFs are among the most widely used machine learning algorithms due to their robustness and versatility. Similar to that of DT, the construction of RF classifiers involved using the RF package from Python's scikit-learn module [52].

### 2.6 Performance Evaluation

The performance of the classifier was evaluated by using the weighted F1 score [55-64]. This metric is a variant of the macro F1 score and is designed to take into account imbalances in sample size. The calculation of the weighted F1 involves assigning weights proportional to the sample size of each class. The specific formula for this metric is:


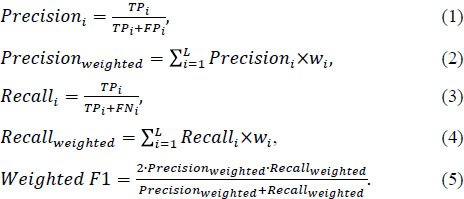


In this formula, *i* signifies one class, *w_i_* represents the ratio of the samples in each class to the total sample size, and *L* stands for the number of classes. Furthermore, *TP* corresponds to the true positives, *FP* stands for the false positives, and *FN* refers to the false negatives.

In addition, we employed two classic measurements to display the performance of classifiers fully, including classification accuracy (ACC) and Matthews correlation coefficient (MCC) [65-67]. ACC is the most widely used measurement. It is defined as the proportion of correctly predicted samples among all samples. When the dataset is imbalanced, ACC is not a perfect measurement. In this case, MCC is objective. For the computation of this measurement, two matrices *X* and *Y* are created in advance, wherein *X* stores the true classes of samples, whereas *Y* collects the predicted classes of samples. Accordingly, MCC is computed by using the formula


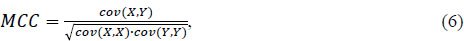


where *cov(X,Y)* stands for the correlation coefficient of *X* and *Y*.

## 3 RESULTS

Fig. (**1**) presents the workflow of our study, wherein we leveraged various machine learning algorithms. Our exhaustive scientific investigation involved analyzing distinct samples of gut bacteria, including *Actinobacteria*, *Bacteroidetes*, and *Firmicutes*, by using these algorithms. We initiated the analysis with 11 diverse feature ranking algorithms, ensuring a meticulous and robust analysis. Subsequently, we employed IFS to discern crucial biomarkers within our dataset. In the culmination stage, we amalgamated IFS with a classification algorithm, enabling us to pinpoint pivotal features correlated with gut bacteria and ultimately facilitating the development of a rigorous quantitative classification system. The feature names corresponding to the feature IDs are listed in Table **S1**.

### 3.1 Feature Ranking Results

In this study, we employed 11 distinct feature ranking algorithms—AdaBoost, CatBoost, ExtraTrees, Lasso, LightGBM, MCFS, mRMR, RF_ZL, Ridge, SVM-order, and XGBoost—to discern the key features associated with gut bacteria. Each of these algorithms generated a feature list, cumulatively incorporating 993 features for further analysis. Our study exhaustively examined these 993 features across 1478 diverse intestinal bacterial types. A comprehensive compilation of these individual feature lists can be found in Table **S2**.

### 3.2 IFS Results and Feature Intersections

We employed a total of 11 distinct feature ranking algorithms to identify the key features that typify and function within gut bacteria. Each algorithm yielded a list of pertinent features. Subsequently, an IFS method with a stride of 1 was leveraged to derive a feature subset. To guarantee the completeness of the classification, we utilized the weighted F1 metric to compare the performance of two classifiers: RF and DT. The amalgamated evaluation results, calculated by Eqs. 1-6, of these classification algorithms can be found in Table **S3**. Figs. **S1–S3** graphically depict the IFS outcomes, and Fig. (**2**) presents a comparative illustration of performance across the three classes.

Figs. **S1–S3** present the results of evaluating the performance of various machine learning algorithms in classification tasks on the basis of their weighted F1 scores and the number of features they utilized. Top-performing classifiers included DT using 54 features on the AdaBoost feature list (weighted F1 score of 1.000), RF using 31 features on the CATboost feature list (weighted F1 score of 0.999), and RF using 42 features on the ExtraTrees feature list (weighted F1 score of 1.000). Other top-performing classifiers on Lasso, LightGBM, MCFS, mRMR, RF_ZL, Ridge, SVM-order, and XGBoost feature lists applied RF, producing weighted F1 scores of 0.999 to 1.000 by using between 20 and 779 features. The assessment outcomes revealed RF as the optimal classifier for the feature lists generated by all algorithms, except AdaBoost, which favored DT (Figs. **S1–S3**). Table **1** lists the detailed performance of these classifiers, including ACC, MCC, macro F1, and weighted F1. Meanwhile, Fig. (**2**) presents the performance of the best RF and DT classifiers across three intestinal bacterial categories. The RF algorithm exhibited superior performance across all feature lists and groups, with the DT algorithm showing a slight advantage over RF within the second and third classes of the AdaBoost feature list. However, this discrepancy was not statistically significant. These findings underscore the importance of utilizing a diverse array of feature ranking algorithms and classifiers to identify the key features associated with gut bacteria.

It can be observed from Table **1** that the best classifiers on all feature lists yielded a weighted F1of at least 0.999. In Chen *et al.*’s study [33], the best classifier reported the weighted F1 of 0.998, which was lower than those of the best classifiers constructed in this study. At this point, our classifiers were superior to Chen *et al.*’s classifier.

In this study, we utilized the weighted F1 score as the performance metric for classification tasks. The most pertinent feature subsets derived from the Adaboost, CATboost, ExtraTrees, Lasso, LightGBM, MCFS, mRMR, RF_ZL, Ridge, SVM-order, and XGBoost feature lists contained the top 54, 31, 42, 779, 90, 97, 137, 32, 127, 657, and 20 features, respectively. Some feature subsets were quite large (*e.g*., the feature subsets on the Lasso feature list consisted of 779 features) and were not useful for further investigation. In view of this situation, extracting the most essential features from this large subset was necessary. These essential features should satisfy the following requirements: (1) considerably fewer features than the original feature subsets, and (2) provide the classifier with high performance. Therefore, we extracted the most essential features from the subsets with numerous features. The number of these features and the performance of the classifier with these features are listed in Figs. **S1–S3**. For example, on the Lasso feature list, the original feature subset contained 779 features. After careful observation, we found that when the top 13 features were adopted, the RF classifier can yield the weighted F1 score of 0.994, which was slightly lower than 0.999, by using the original feature subset. After such operations, we extracted a few features on each feature list containing the top 12, 7, 19, 13, 17, 26, 31, 10, 18, 412, and 20 features on Adaboost, CATboost, ExtraTrees, Lasso, LightGBM, MCFS, mRMR, RF_ZL, Ridge, SVM-order, and XGBoost feature lists, respectively. We conducted an intersection analysis of these critical feature subsets to explore the commonalities and differences among the above features and subsequently visualized the commonality of these subsets *via* the Venn diagram displayed in Fig. (**3A**). Fig. (**3B**) illustrates the intersection of the top 10 features from the 11 ordered feature sets. Detailed data regarding the intersections of feature subsets are provided in Tables **S4** and **S5**. By identifying features recurring across multiple feature subgroups, we underline their importance in the behavioral patterns of intestinal bacteria. These features have proven to be valuable for studies involving intestinal bacteria.

The central biological functions of these features are integral to the comprehension and investigation of gut bacteria. We have analyzed these functions extensively in the "Discussion" section.

### 3.3 Classification Rules for Identifying Actinobacteria, Bacteroidetes, and Firmicutes

This study utilized a DT model, a type of white-box machine learning model, to establish classification rules. Rules facilitated the identification of specific types of gut bacteria, particularly *Actinobacteria*, *Bacteroidetes*, and *Firmicutes*. A detailed list of these rules can be found in Table **S5**. These rules were developed by using feature sets derived from 11 unique feature ranking algorithms. As illustrated in Fig. (**4**), among the various feature ranking algorithms, the feature subsets rooted in Lasso and Ridge generated the highest number of rules for identifying *Actinobacteria*, producing eight rules. Conversely, the ExtraTrees feature set yielded the fewest rules, with only four rules. On the basis of all 11 feature subsets, the rules for identifying *Bacteroidetes* all resulted in a single rule. For the identification of *Firmicutes*, seven rules were generated on the basis of the ExtraTrees, Lasso, RF_ZL, and Ridge feature subsets, whereas the CATboost, LightGBM, mRMR, and SVM-order feature subsets produced the lowest number of rules, with five each. Minimal variation was found in the number of rules created for identifying different types of gut bacteria, with most rules aimed at *Actinobacteria* and *Firmicutes* and *Bacteroidetes* having the lowest number of rules. In the "Discussion" section, we further delve into the crucial features that may offer nuanced insights for the identification and research of intestinal bacteria.

## 4 DISCUSSION

### 4.1 Analysis of Highly Ranked Features in 11 Feature Ranking Algorithms

Our study is a reanalysis of the work performed by Chen *et al.* Existing studies primarily use experimental methods, such as 16S rRNA sequencing, metagenomic sequencing, and gene amplicon sequencing, to differentiate gut microbiota. These studies often focus on the distribution of bacterial phyla, such as *Firmicutes*, *Bacteroidetes*, and *Actinobacteria*, in response to factors like diet, health conditions, and treatments. For example, diet has been shown to have a stronger effect than exercise on gut microbiome composition [68], and it significantly influences the abundance of *Firmicutes* and *Bacteroidetes* [69, 70]. Antibiotic treatment can disrupt these phyla [71], while probiotics help restore the balance [72]. Additionally, geographical factors and diet contribute to variations in microbiota composition [73]. Experimental techniques like 16S rRNA sequencing have proven effective in profiling microbial communities, but they often fall short in identifying functional biomarkers that can provide deeper insights into the regulatory roles of gut microbiota. Our study addresses this gap by introducing machine learning-based feature ranking methods that allow for the rapid identification of key features that distinguish specific gut bacteria, offering a more efficient approach compared to traditional experimental techniques. Although experimental methods have been the primary focus, machine-learning techniques for differentiating gut bacteria are still scarce. In contrast to traditional approaches, which rely heavily on sequencing and taxonomic classification, our research introduces a novel machine-learning approach to identifying the most important functional features of gut bacteria. Chen *et al.*’s study, for instance, focused on taxonomic diversity and did not explore machine learning methods for identifying key biomarkers. We applied eleven feature ranking algorithms to identify features that are critical for distinguishing between bacterial phyla, providing a more scalable and objective framework for microbiome research. Our primary objective is to identify the most important features associated with different phyla of gut bacteria. By applying these algorithms, we obtained 11 ordered feature lists, which consistently highlighted the key features deemed important across these lists. These features are known to be linked to intestinal bacteria or specific bacterial strains, and their value has been previously established in scientific literature. Our findings contribute to an updated and comprehensive understanding of the characteristics and functions of intestinal bacteria. Furthermore, our results offer insights into the microstructure of gut microbiota and deepen our understanding of the regulatory role of bacterial subpopulations within the human intestinal tract. Some top features are provided in Table **2**. In Chen *et al.*’s study, 25 key features were extracted. Among features listed in Table **2**, none of them were identified in Chen *et al.*’s study, indicating the novel findings in this study.

The first identified key feature, F_846, corresponds to VFG000077, sourced from the VFDB. VFG000077 is representative of ATP-dependent Clp proteases, proteolytic subunit (ClpP) forming the core of the bacterial protein-degradation machinery. The ClpP protease is well conserved between bacteria and eukarya, and it plays a vital role in the degradation of proteins and quality control after degradation or under stress conditions by selective elimination of misfolded or damaged proteins, possibly in concert with an ATPase such as ClpA or ClpX [74]. The exact functions of ClpP proteases are enormously different between *Firmicutes*, *Bacteroidetes*, and *Actinobacteria* [75, 76]. In *Firmicutes*, ClpP is involved in stress adaptation and protein quality control, presumably conferring increased cell survival under harsh conditions [77]. ClpP in *Bacteroidetes* would likely be associated with maintaining the protein homeostasis necessitated within the fluctuating nutrient landscape of the gut [78, 79]. In *Actinobacteria*, ClpP controls the generation of complex secondary metabolites necessary for adapting to fearsome growth conditions [80]. Thus, the diversified functions of ClpP proteases in these bacterial phyla reflect their diversified ecological niches and different physiological requirements.

The next key feature, F_412, is *Staphylococcus aureus* gyrB, conferring resistance to aminocoumarin, sourced from the ARDB. The gyrB gene presented as a significant marker, guaranteeing the difference of *Actinobacteria*, *Bacteroidetes*, and *Firmicutes* due to peculiarities, and has a better resolution on the level of species and subspecies compared to 16S rDNA, where only minor parE gene interference during the case of *Firmicutes* is reported. A research paper by Poirier *et al.* on analyzing the bacterial diversity in the meat spoilage microbiota and seafood spoilage microbiota, using the gyrB amplicon sequencing, showed that this gene could help differentiate between the groups [81]. The GyrB is also based on differential expression in *Actinobacteria*, *Bacteroidetes*, *Firmicutes*, and another phylum, thus establishing itself as the best beacon for differentiation of these phyla, at least during the process of swine manure composting. *Bacteroidetes* dominate the gyrB-based profiles for the mesophilic group, while *Firmicutes*, along with Proteobacteria, contribute to the profiles at the thermophilic and cooling stages, respectively [82]. The variations in the gyrB, often linked to resistance against aminocoumarin antibiotics, present in *Actinobacteria*, make these bacteria unique from other bacterial groups [83]. Similarly, in *Firmicutes* and *Bacteroidetes*, the gyrB gene variations contribute to the overall resistance profile against aminocoumarin antibiotics [84]. Therefore, the F_412 gene is indeed helpful for the differentiation of bacterial communities and their resistance profiles.

Another significant feature, F_579, represents "vanRC", sourced from the CARD. The vanRC gene is carried by the van gene clusters. It is responsible for regulating vancomycin resistance in general, particularly in bacteria, this one being among Gram-positive species. *Firmicutes*, including *Staphylococcus aureus* and *Enterococcus* species, depend on the modification of the cell wall precursor from D-Ala-D-Ala to D-Ala-D-Lac to induce vancomycin resistance, which is again controlled by van gene clusters with vanA, vanB, vanD, vanM, and vanN, which are transferred independently and controlled by the vanRC gene [85, 86]. This gene activates the expression of resistance genes through a two-component system that, therefore, results in a drastic decrease in vancomycin binding and, hence, its efficiency [86]. The expression of vans is likely to be much higher in the gut *Firmicutes* than in the vancomycin-susceptible bacteria, though it's not experimentally shown. Its activity in the *Firmicutes* contributes to their resistance mechanism. For example, in *Actinobacteria*, *Bifidobacterium*, and *Corynebacterium*, the expression of the vanRC gene is usually minor or absent. This is because the bacteria are not commonly in contact with vancomycin under normal gut conditions [87, 88]. *Bacteroidetes* contribute a vital role in the gut ecosystem by degrading dietary fibers and other complex carbohydrates, and they rely more on the environmental utilization of nutrients and metabolic flexibility—capabilities of the bacterial community than on vancomycin resistance [27]. The presence of van RC and its activity level will thus differentiate these three types of intestinal bacteria.

The following crucial feature, F_798, pertains to the "Metabolism: Overview: Fatty acid metabolism". Fatty acid metabolism is fundamental in bacterial cell structure and energy storage, significantly impacting their survival and function. This metabolic pathway is crucial for *Firmicutes*, which are known for their ability to ferment various carbohydrates, leading to extensive fatty acid production [89, 90]. In contrast, *Actinobacteria* employ fatty acid metabolism differently, often focusing on the synthesis of complex lipids necessary for their resilient cell walls, which is critical in their role as decomposers in various ecosystems [89]. Meanwhile, *Bacteroidetes* demonstrate a unique approach to fatty acid metabolism, adapting their pathways to optimize energy extraction from polysaccharides commonly found in the human gut [91]. Thus, variations in fatty acid metabolism pathways can significantly aid in distinguishing these gut bacteria.

The last key feature, F_718, corresponds to " Metabolism: Carbohydrate metabolism: C5-Branched dibasic acid metabolism". The C5-branched dibasic acid metabolism pathway involves the catabolism of branched-chain amino acids, which are crucial for the synthesis of branched-chain fatty acids used in the bacterial cell wall. *Actinobacteria* are particularly adept at utilizing this pathway to produce mycolic acids, a type of branched fatty acid that is a major component of their cell walls, providing chemical resilience and pathogenic resistance [89, 92]. *Firmicutes*, on the other hand, might incorporate elements of this pathway differently to support their diverse metabolic capacities in fermentative environments [90]. *Bacteroidetes* appear to have a reduced reliance on this pathway, reflecting their adaptation to the nutrient-rich conditions of the human gut, where simpler metabolic routes are favored [91]. This metabolic distinction is vital for understanding the ecological niches and interaction dynamics of these bacteria in the gut microbiome.

Based on our newly discovered key features, we have summarized the differences among the three gut bacteria as follows: Metabolic traits of *Actinobacteria*, *Bacteroidetes*, and *Firmicutes* are the outcome of niche specialization taken by this group. *Firmicutes* use ClpP proteases for stress adaptation and protein quality control. *Bacteroidetes* use it to maintain protein homeostasis in the gut, while *Actinobacteria* use it to produce complex secondary metabolites. The additional edge for this division could be the gyrB gene, which is associated with antibiotic resistance. On the other hand, high expression of vanRC leading to vancomycin resistance is found in *Firmicutes*, with very minimal or no amount of expression in *Actinobacteria* and *Bacteroidetes*. The variation in fatty acid metabolism reflects this functional diversity: *Firmicutes* are fermenters of carbohydrates, *Actinobacteria* synthesize complex lipids for their cell walls, and *Bacteroidetes* extract energy from gut polysaccharides. Finally, c5-branched dibasic acid metabolism reveals that *Actinobacteria* produce mycolic acids to reinforce cell walls, *Firmicutes* support many metabolic activities, and *Bacteroidetes* favor more straightforward routes in the nutrient-rich gut. Biological differences underlie different physiological requirements and ecological roles of these groups within the gut microbiome.

We found that although the feature ranking results of the 11 algorithms showed some differences, they all achieved high classification performance (weighted F1 score > 0.99) based on their respective optimal feature subsets. These differences can be attributed to the diverse principles, applicable conditions, and preferences for the inherent data patterns of these algorithms. For example, tree-based algorithms, such as RF, can capture nonlinear relationships, whereas linear models, like Lasso, focus on linear feature relationships. Despite these differences, some features (*e.g*., F_412) consistently ranked highly across multiple algorithms, supporting their importance from different perspectives. Moreover, the high classification performance achieved by all of the algorithms demonstrates the effectiveness of their selected features in distinguishing bacterial taxa. In summary, the comprehensive analysis of multiple algorithms in our study provides a more robust and reliable understanding of the functional characteristics of gut bacteria than the single-algorithm analysis used by Chen *et al.* The differences in algorithmic performance results highlight the necessity and advantages of our multialgorithm approach.

The features identified in our work have important applications in understanding the gut microbiome. They can aid in identifying specific bacterial taxa or functional groups, identifying the functional profiles of the gut microbiota, and guiding personalized interventions, such as dietary modulation or targeted probiotic therapy. Compared with traditional sequencing-based methods, our functional annotation–based approaches offer a more direct, comprehensive, and scalable approach to characterize the functional capacity and host–microbe interactions of the gut microbiome, thus facilitating the development of novel biomarkers and therapeutic strategies. Some of the key features identified in our study, such as F_846 (ClpP protease), F_412 (gyrB gene), and F_579 (vanRC gene), have either been previously reported in microbiome-related research or represent novel findings that were not explored in depth. For example, the ClpP protease has been identified as playing a crucial role in stress adaptation and protein quality control across different bacterial phyla [93, 94], and the gyrB gene has been shown to differentiate bacterial taxa based on antibiotic resistance profiles [95, 96]. However, the specific roles of these features in gut microbiota differentiation have not been thoroughly investigated in the context of microbiome-based machine learning. Our study links these functional markers with bacterial phyla differentiation, offering new insights into the ecological roles of gut bacteria. In contrast to traditional sequencing-based methods, our machine learning framework provides a more direct, comprehensive, and scalable approach to analyzing the functional capacity of the gut microbiome, facilitating the development of novel biomarkers and therapeutic strategies.

### 4.2 Analysis of Decision Rules for Identifying Actinobacteria, Bacteroidetes, and Firmicutes

In this study, we employed two classification algorithms, namely, RF and DT, to derive quantitative rules from 11 distinct feature sets. The expression patterns of pivotal features facilitated the identification of three gut bacterial types: *Actinobacteria*, *Bacteroidetes*, and *Firmicutes*. A detailed analysis of critical parameters, depicted in Table **3**, supports these rules. The values in Table **3** indicate the performance of the features. Recent literature corroborates the reliability of our established parameters and provides a comprehensive elucidation of their importance. Our research enhances the understanding of these bacterial types and underscores the potential of machine learning–based rules in the identification of intestinal bacteria, thereby paving the way for novel perspectives in gut health–related microbiology research.

The first rule, **Rule 0**, facilitates the identification of *Actinobacteria* based on two specific parameters: F_618 and F_624. The F_618 parameter, which is synonymous with the "AMPK signaling pathway," is expressed at a low level in this rule and serves as a determinant for *Actinobacteria*. Certain gut bacteria are known to ferment dietary fiber into short-chain fatty acids, thereby influencing the AMPK signaling pathway. For example, certain species of *Bifidobacterium* generate acetate, which is subsequently converted into butyrate by other gut bacteria. Chronic butyrate exposure has been linked to the downregulation of the AMPK signaling pathway [97]. Additionally, *Bifidobacterium animalis* subsp. *lactis* SF has been reported to downregulate the AMPK signaling pathway [98], underscoring the potential of F_618 as a marker for *Actinobacteria* identification. The next parameter, F_624, represents the "PI3K–Akt signaling pathway" and is also downregulated in **Rule 0**. *Bifidobacterium* species have a widespread association with the regulation of the PI3K–Akt signaling pathway. In particular, *Bifidobacterium animalis* subsp. *lactis* SF, classified under *Actinobacteria*, has been found to downregulate the PI3K–Akt signaling pathway [97]. Furthermore, a concurrent downregulation of the PI3K–Akt signaling pathway has been reported in *Bifidobacterium lactis* and *Lactobacillus plantarum* [99]. *Bifidobacterium infantis* has also been associated with inhibiting the PI3K–Akt-mTOR signaling pathway [100], supporting the utility of low F_624 expression as a characteristic indicator of *Actinobacteria*.

**Rule 1** serves as a tool for identifying *Bacteroidetes* and encompasses three parameters: F_823, F_618, and F_624. The first of these, F_823, symbolizing "protein digestion and absorption," shows an upregulation in this rule. Past studies have analyzed the relationship between gut bacteria and protein digestion and absorption, revealing that *Bacteroidetes* have a pronounced ability to degrade proteins and complex carbohydrates [101, 102]. In particular, the *Bacteroidetes* genus *Prevotella* substantially contributes to protein degradation, especially in the rumen [103, 104]. However, additional research is required to delineate the unique protein degradation capabilities of *Bacteroidetes* over other bacterial groups. Next, in a reversal from its role in *Actinobacteria* identification, the F_618 parameter is upregulated in **Rule 1**. *Bacteroides thetaiotaomicron*, for example, has been found to reverse alcohol-induced AMPK inhibition [105], and oleic acid from the same species can augment AMPK expression in macrophages [106]. Furthermore, several *Bacteroidetes*, like *Bacteroides* spp., are known propionate producers [107, 108], a compound associated with AMPK pathway activation [109, 110]. Therefore, the upregulation of F_618 could aid in identifying *Bacteroidetes*. Lastly, the F_624 parameter is upregulated in **Rule 1**, indicating a potential link to the PI3K–Akt signaling pathway. *Bacteroides fragilis*, for example, has been found to counteract methotrexate-induced butyrate reductions, thereby regulating the PI3K–Akt signaling pathway [111]. Additionally, the *Muribaculaceae* family, previously classified under *Bacteroidetes*, was positively correlated with PI3K–Akt signaling pathway activation [112]. Moreover, *Bacteroidetes*-produced sphingolipids have been associated with the mediation of the PI3K–Akt pathway [113]. Collectively, these findings suggest that F_624 upregulation could enhance the accurate identification of *Bacteroidetes*.

The final rule, **Rule 2**, is utilized to identify *Firmicutes* and comprises three parameters: F_617, F_798, and F_731. In this rule, the first parameter, F_617, refers to the phosphotransferase system (PTS) and is upregulated. PTS is a crucial mechanism for the survival and colonization of gut bacteria, enabling the efficient utilization of nutrients, including complex sugars that may be inaccessible to other organisms [114, 115]. For instance, a glucose-specific PTS was identified in *Clostridium acetobutylicum* [116]. The primary hexose transport system of this species is phosphoenolpyruvate-dependent PTS [117]. Moreover, genome sequencing revealed that 46 out of 277 genes in *Lactobacillus pentosus* encode for PTS [118]. Notably, PTS plays a role in the virulence regulation of certain *Firmicutes*, such as *Streptococcus pneumoniae*, wherein mannose PTS regulates virulence determinant genes [119]. Therefore, F_617 could be instrumental in identifying *Firmicutes*. The next parameter, F_798, pertains to "fatty acid metabolism," exhibiting high expression in **Rule 2** for *Firmicutes* identification. To illustrate, milk fermented by *Lactobacillus rhamnosus* hsryfm 1301 has been linked to accelerated fatty acid metabolism and triglyceride hydrolysis [120]. Likewise, *Clostridium butyricum* MIYAIRI 588 has been associated with increased fatty acid metabolism [121], highlighting F_798's potential utility in identifying *Firmicutes*. Finally, F_731 represents "carbon fixation pathways in prokaryotes," showing high expression in **Rule 2**. Despite the prevalence of carbon fixation pathways in autotrophic bacteria, *Firmicutes*, along with *Actinobacteria* and *Bacteroidetes*, are predominantly heterotrophic [122]. Nevertheless, two *Firmicutes* members, *Clostridium autoethanogenum* and *Clostridium ljungdahlii*, are involved in the Wood–Ljungdahl pathway, which facilitates the conversion of carbon dioxide into acetyl coenzyme A for various cellular processes [123, 124]. Further investigations are needed to elucidate the role of *Firmicutes* in intestinal carbon fixation. F_731 may prove beneficial for *Firmicutes* identification.

### 4.3 Limitations of this Study

This study successfully identified some key features that can indicate the differences of *Actinobacteria*, *Bacteroidetes*, and *Firmicutes*. However, some limitations also exist. First, several feature selection methods were employed to identify important features. However, we still cannot know whether they were enough for mining all the important features. Second, this study lacked experimental verification. We only discussed the latent associations between key features or rules and bacterial classification. The wet experiments were still needed to confirm these findings. In future, we will continue this work for improving our understanding of gut bacteria.

## CONCLUSION

 Our research aims to deepen the understanding of gut microbiota, a fundamental factor in human health, by applying sophisticated machine learning methodologies to a dataset comprising 1478 samples, each characterized by 993 unique attributes. Initially, we utilized eleven feature-ranking algorithms (AdaBoost, CatBoost, ExtraTrees, Lasso, LightGBM, MCFS, mRMR, RF_ZL, Ridge, SVM-order, and XGBoost) to identify features on the basis of their importance, culminating in an ordered subset of 11 key features. By integrating an IFS approach within our computational structure, which contained two classification algorithms and SMOTE, we successfully identified the features critical for accurate classification. Compared with one previous computational analysis developed by Chen *et al.*, five key features (Table **2**) that were exclusively identified in this study were found to be associated with functional differences among gut bacterial phyla, such as *Actinobacteria*, *Bacteroidetes*, and *Firmicutes*. Considering that a single feature may be insufficient to distinguish bacterial taxa at the species level definitively, rules (Table **3**), the combinations of multiple key features could provide a comprehensive and detailed characterization of different bacterial groups. Furthermore, our research effectively established classifiers for accurately identifying *Actinobacteria*, *Bacteroidetes*, and *Firmicutes* by using two classification algorithms (RF and DT). Through comprehensive analysis with multiple advanced algorithms, our work revealed the differences in the functional characteristics of intestinal bacteria more comprehensively than the study of Chen *et al.* and proposed a set of effective bacterial classification methods. Our research not only deepens our understanding of intestinal flora, it also provides new ideas and tools for future research. Although individual features may not completely distinguish bacterial taxa, our results demonstrate that feature combination analysis is a promising approach for elucidating the functional profiles and ecological relationships within the gut microbiome. In summary, our study emphasizes the fundamental role of machine learning in gut microbiota research, enriching our understanding of the intricate functions of gut bacteria. It also provides a robust groundwork for future exploration in this dynamic and rapidly developing field, heralding advancements in human health and well-being.

## Figures and Tables

**Fig. (1):**
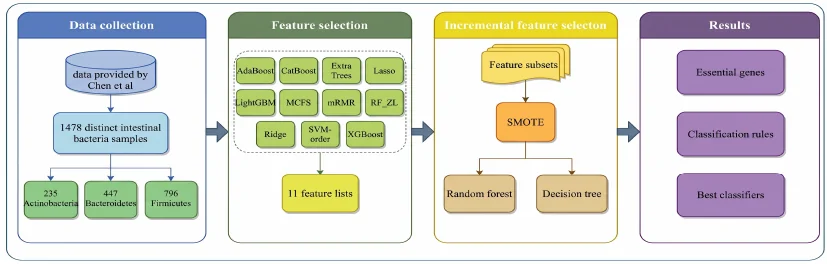
Flow chart of the entire analysis process. Gene profile data from 1478 gut bacterial samples were analyzed. Eleven feature ranking algorithms were used to generate ordered feature lists on the basis of features’ correlation with gut bacteria and incorporated into the IFS framework. The classification model was constructed to identify specific gut bacterial species, and the best feature subsets and classification rules were extracted.

**Fig. (2):**
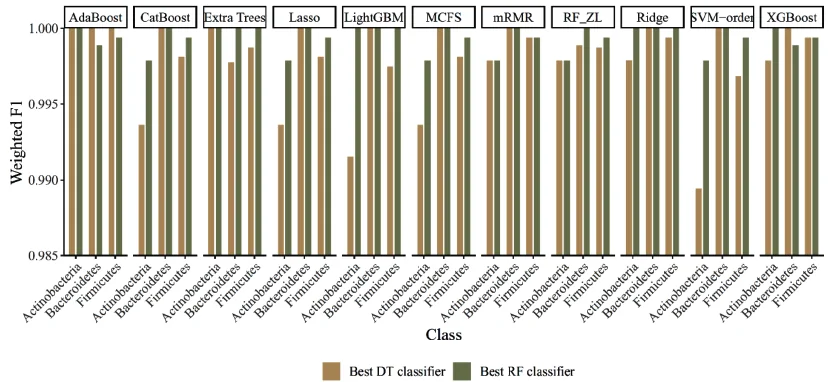
Performance of the optimal models built on 11 feature lists in three classes. A grouped bar chart was utilized to compare the performance of two classifiers (RF and DT) between three classes for each feature set with the weighted F1 score as the criterion.

**Fig. (3):**
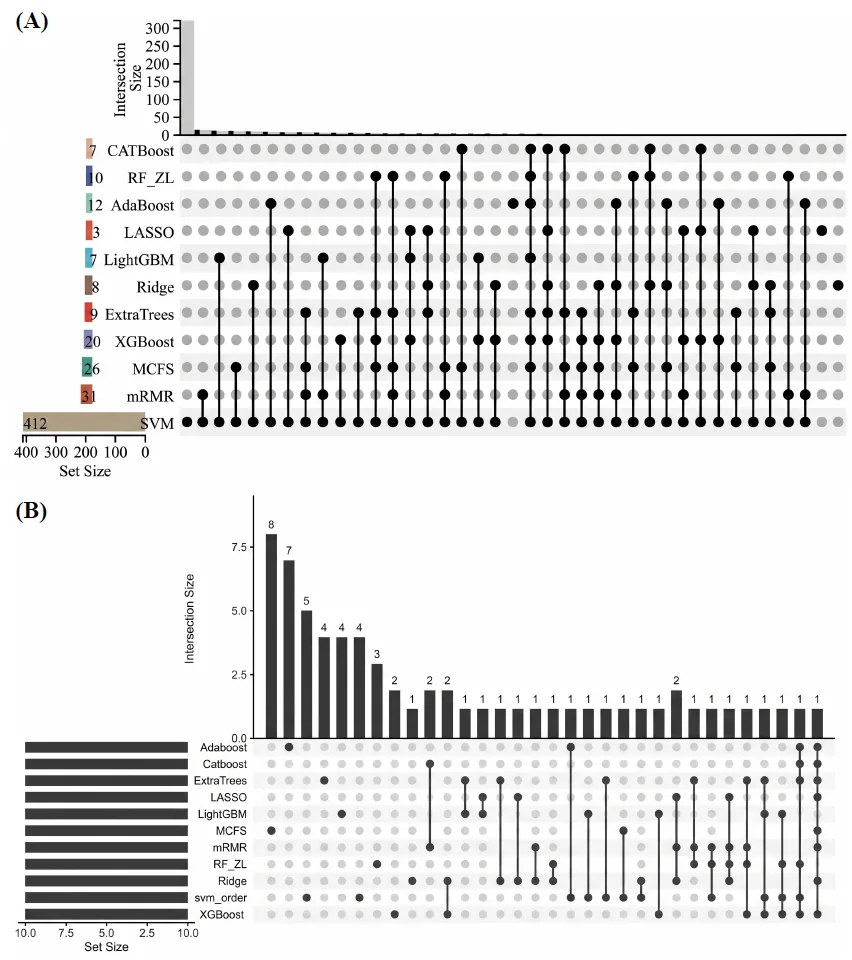
UpSet Venn diagrams of the best subset offeatures obtained by using 11 feature sorting algorithms.**A**: UpSet Venn diagram based on 11 optimal feature subsets.**B**: UpSet Venn diagrams based on the top 10 features of 11feature subsets. "Set Size" is the count of the number offeatures in each set. "Intersection Size" is the count of thenumber of features after taking the intersection of the seven featuresets. Black dots indicate the unique features of a feature set, and thelines between dots indicate the unique intersection of different featuresets.

**Fig. (4):**
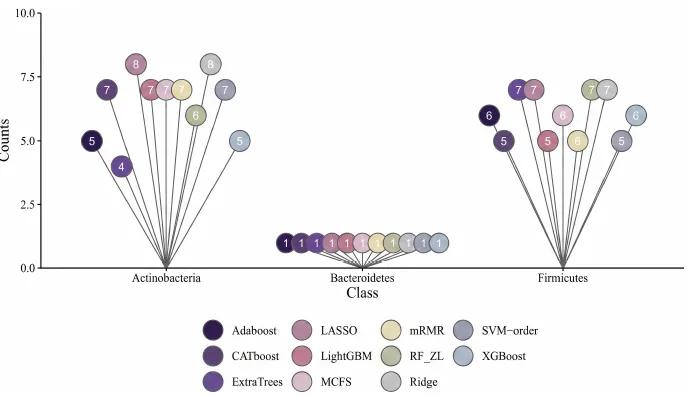
Lollipop plot showing the number of rules for *Actinobacteria*, *Bacteroidetes*, and *Firmicutes* classes. The number of rules obtained by DT based on 11 different feature subsets in the three predictive classes is shown. Different colors represent the different feature lists obtained by the 11 feature sorting algorithms.

**Table 1:** Performance of the best classifiers on different feature lists.

**Feature List**	**Classification Algorithm**	**Number of Features**	**ACC**	**MCC**	**Macro F1**	**Weighted F1**
AdaBoost feature list	DT	54	1.000	1.000	1.000	1.000
CatBoost feature list	RF	31	0.999	0.999	0.999	0.999
ExtraTrees feature list	RF	42	1.000	1.000	1.000	1.000
Lasso feature list	RF	779	0.999	0.999	0.999	0.999
LightGBM feature list	RF	90	1.000	1.000	1.000	1.000
MCFS feature list	RF	97	0.999	0.999	0.999	0.999
mRMR feature list	RF	137	0.999	0.999	0.999	0.999
RF_ZL feature list	RF	32	0.999	0.999	0.999	0.999
Ridge feature list	RF	127	1.000	1.000	1.000	1.000
SVM-order feature list	RF	657	0.999	0.999	0.999	0.999
XGBoost feature list	RF	20	0.999	0.999	0.999	0.999

**Table 2:** Essential features associated with gut bacteria identified by feature ranking algorithms.

**Feature**	**Feature Name**
F_846 (VFDB)	VFG000077(ATP-dependent Clp protease proteolytic subunit)
F_412 (ARDB)	*Staphylococcus aureus* gyrB conferring resistance to aminocoumarin
F_579 (CARD)	vanRC
F_798 (KEGG)	Metabolism: Overview: Fatty acid metabolism
F_718 (KEGG)	Metabolism: Carbohydrate metabolism: C5-Branched dibasic acid metabolism

**Table 3:** Representative rules for identifying actinobacteria, bacteroidetes, and firmicutes.

**Rules**	**Parameters**	**Predicted Class**
Rule 0	F_618 (Environmental information processing: Signal transduction: AMPK signaling pathway) (KEGG) ≤ 0.5	*Actinobacteria*
	F_624 (Environmental information processing: Signal transduction: PI3K–Akt signaling pathway) (KEGG) ≤ 0.5	
Rule 1	F_823 (Organismal systems: Digestive system: Protein digestion and absorption) (KEGG) > 0.5	*Bacteroidetes*
	F_618 (Environmental information processing: Signal transduction: AMPK signaling pathway) (KEGG) > 0.5	
	F_624 (Environmental information processing: Signal transduction: PI3K–Akt signaling pathway) (KEGG) > 0.5	
Rule 2	F_617 (Environmental information processing: Membrane transport: Phosphotransferase system) (KEGG) > 0.5	*Firmicutes*
	F_798 (Metabolism: Overview: Fatty acid metabolism) (KEGG) > 3.5	
	F_731 (Metabolism: Energy metabolism: Carbon fixation pathways in prokaryotes) (KEGG) > 9.5	

## Data Availability

The datasets supporting the findings of the article are available in the Chen et al.’s study (https://www.frontiersin.org/articles/10.3389/fgene.2019.01146/full (33)).

## LIST OF ABBREVIATIONS

AdaBoost: Adaptive Boosting
ARDB: Antibiotic Resistance Genes Database
CARD: Comprehensive Antibiotic Research Database
CatBoost: Categorical Boosting
DT: Decision Tree
Extra Trees: Extremely Randomized Trees
IFS: Incremental Feature Selection
KEGG: Kyoto Encyclopedia of Genes and Genomes
Lasso: Least Absolute Shrinkage and Selection Operator
LightGBM: Light Gradient Boosting Machine
MCFS: Monte Carlo Feature Selection
mRMR: Minimum Redundancy Maximum Relevance
RF: Random Forest
RF_ZL: Random Forest
Ridge: Ridge Regression
SVM-order: Support Vector Machine - Recursive Feature Elimination
VFDB: Virulence Factors of Pathogenic Bacteria
XGBoost: eXtreme Gradient Boosting
