# Supplementary material for: Identification of Key Features Pivotal to the Characteristics and Functions of Gut Bacteria Taxa through Machine Learning Methods
Source: Curr Gene Ther. 2025 Jul 15;26(4):E15665232367064. doi: 10.2174/0115665232367064250630202337 (PMC13621372; doi:10.2174/0115665232367064250630202337)
Supplement: Supplementary file 1 [file CGT-26-4-E15665232367064_SD1.pdf]

Supplementary Material

Identification of Key Features Pivotal to the Characteristics and Functions of Gut Bacteria Taxa through Machine Learning Methods

ZhanDong Li[1,†], QingLan Ma[2,†], Hao Li[1], Lin Lu[3], Lei Chen[4], Wei Guo[5], KaiYan Feng[6], Tao Huang[7,8,*] and Yu-Dong Cai[2,*]

[1]School of Biological and Food Engineering, Jilin Engineering Normal University, Changchun 130052, China; [2]School of Life Sciences, Shanghai University, Shanghai 200444, China; [3]Department of Radiology, Columbia University Medical Center, New York 10032, USA; [4]College of Information Engineering, Shanghai Maritime University, Shanghai 201306, China; [5]Key Laboratory of Stem Cell Biology, Shanghai Jiao Tong University School of Medicine (SJTUSM) & Shanghai Institutes for Biological Sciences (SIBS), Chinese Academy of Sciences (CAS), Shanghai 200030, China; [6]Department of Computer Science, Guangdong AIB Polytechnic College, Guangzhou 510507, China; [7]Bio-Med Big Data Center, CAS Key Laboratory of Computational Biology, Shanghai Institute of Nutrition and Health, University of Chinese Academy of Sciences, Chinese Academy of Sciences, Shanghai 200031, China; [8]CAS Key Laboratory of Tissue Microenvironment and Tumor, Shanghai Institute of Nutrition and Health, University of Chinese Academy of Sciences, Chinese Academy of Sciences, Shanghai 200031, China

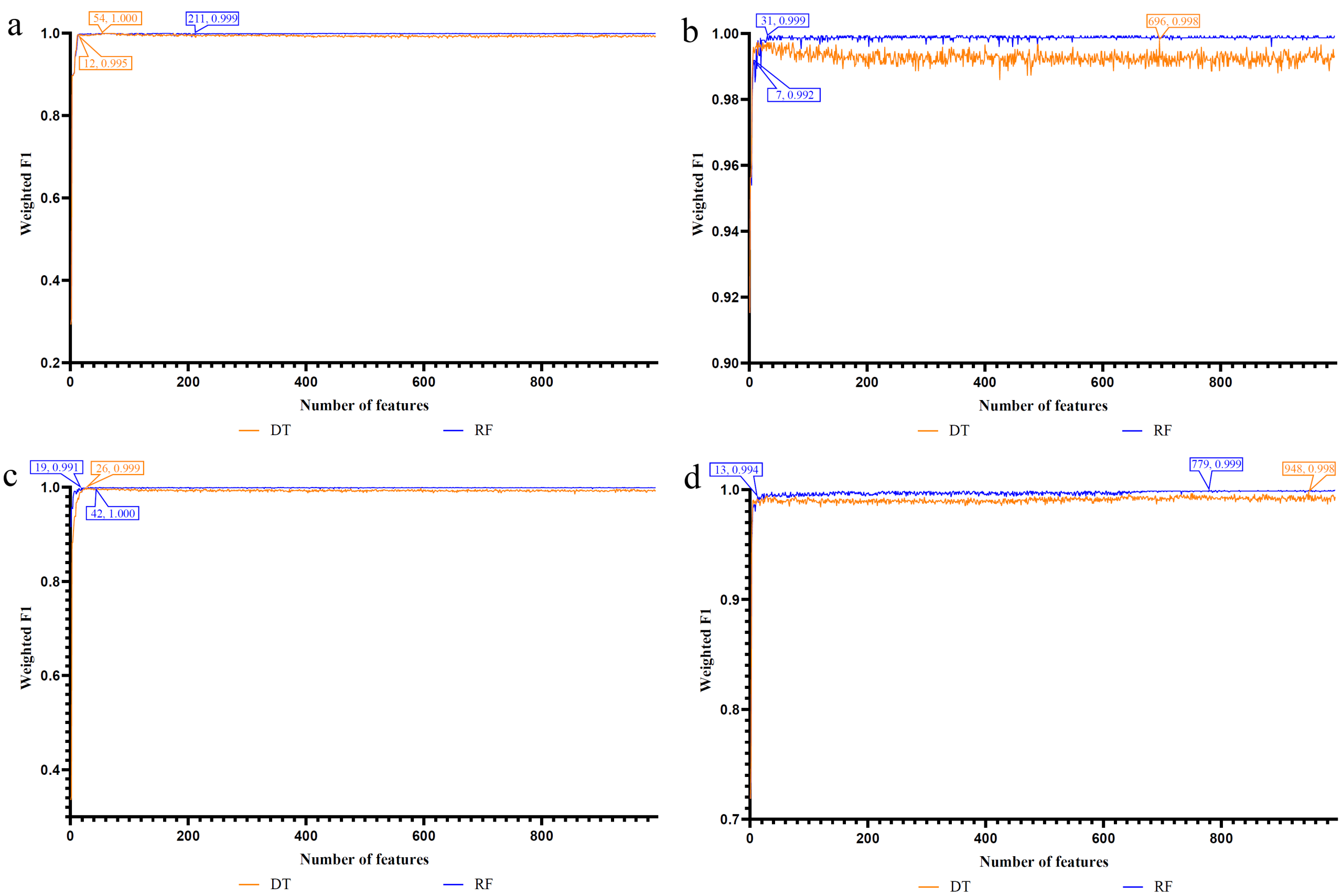

Fig. (S1). IFS curves for evaluating the performance of two classification algorithms on AdaBoost, CatBoost, ExtraTrees, and Lasso feature lists. **a**: IFS curves on AdaBoost feature list. **b**: IFS curves on CatBoost feature list. **c**: IFS curves on ExtraTrees feature list. **d**: IFS curves on Lasso feature list.

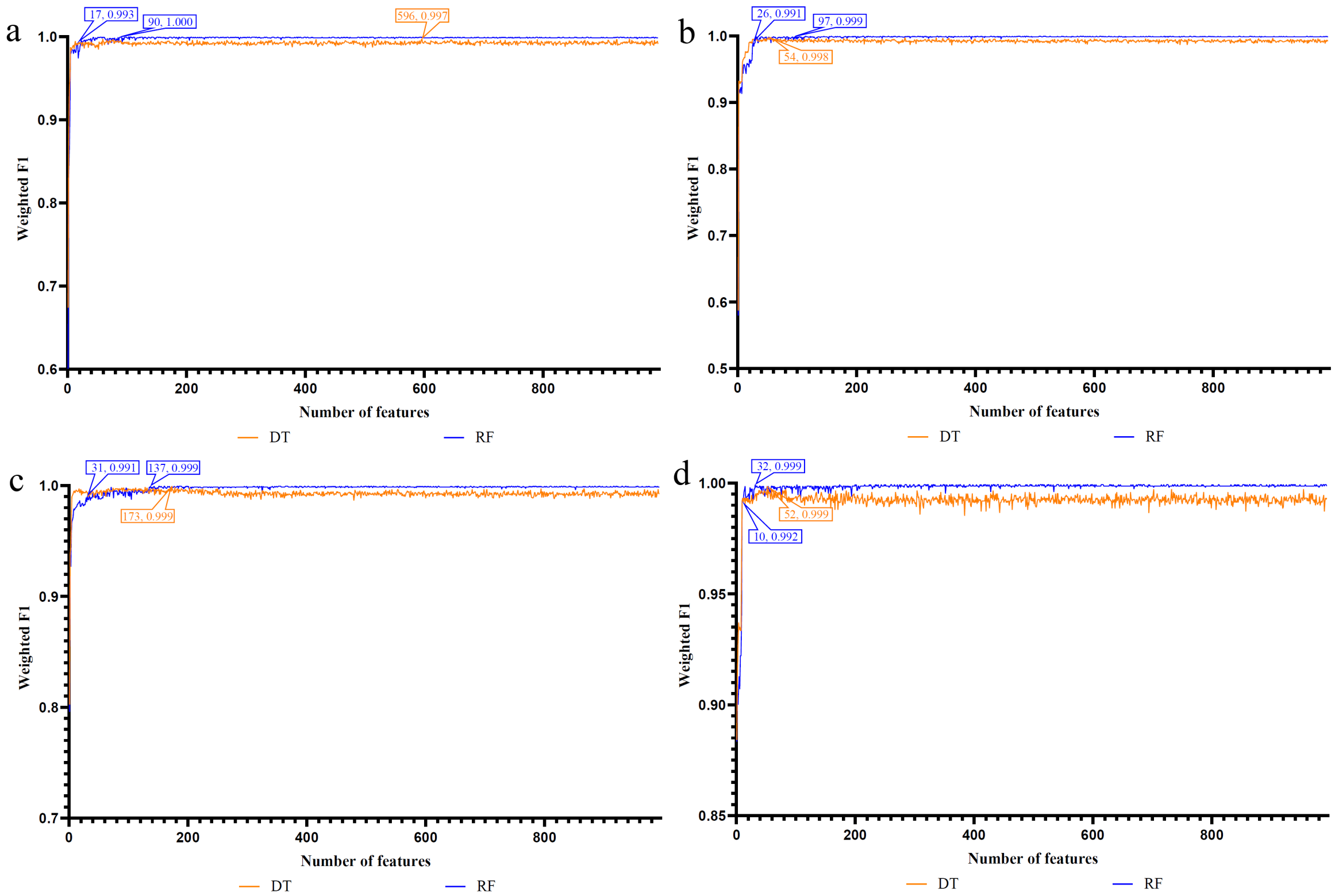

Fig. (S2). IFS curves for evaluating the performance of two classification algorithms on LightGBM, MCFS, mRMR, and RF_ZL feature lists. **a**: IFS curves on LightGBM feature list. **b**: IFS curves on MCFS feature list. **c**: IFS curves on mRMR feature list. **d**: IFS curves on RF_ZL feature list.

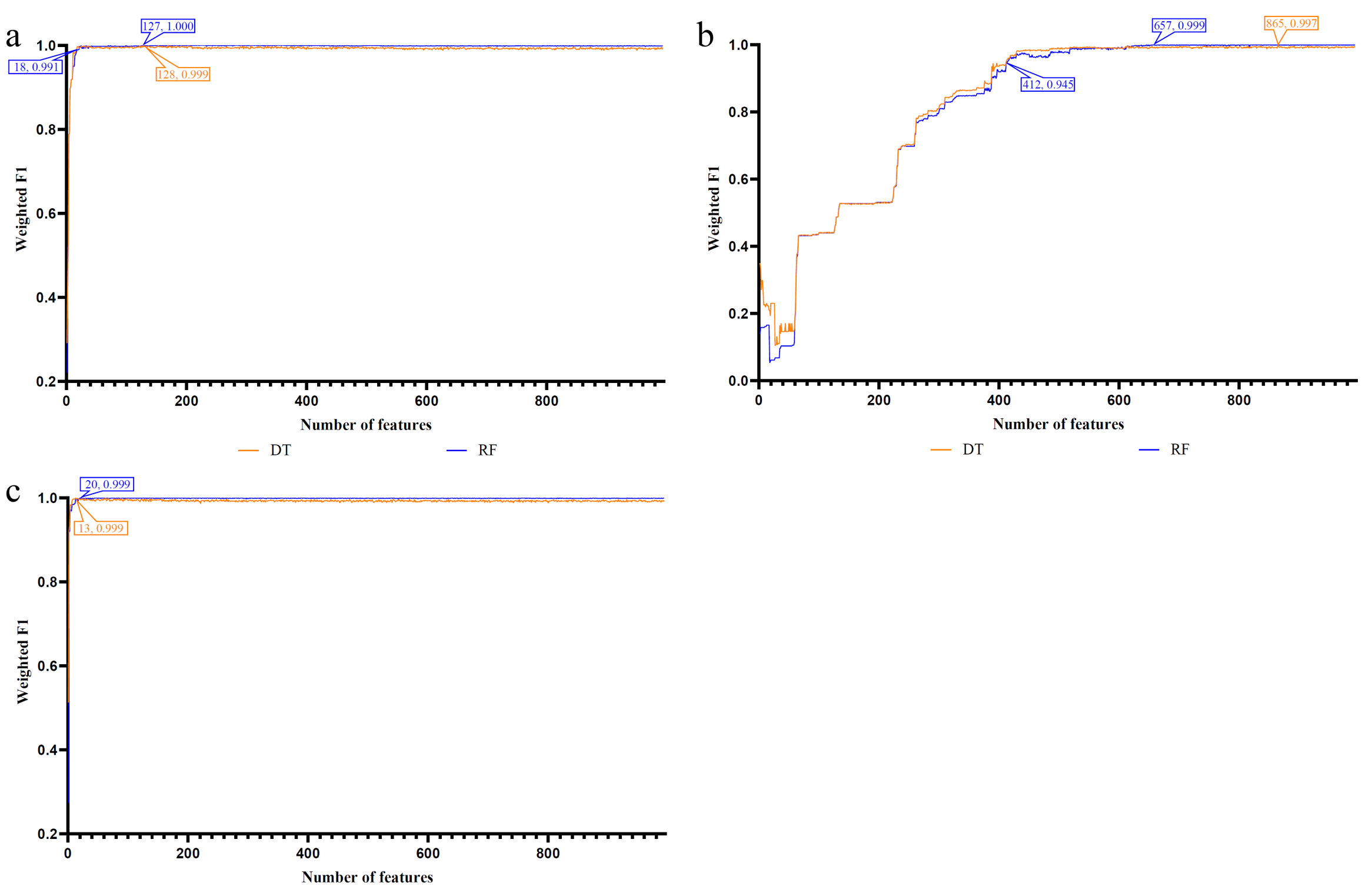

Fig. (S3). IFS curves for evaluating the performance of two classification algorithms on Ridge, SVM-order, and XGBoost feature lists. **a**: IFS curves on Ridge feature list. **b**: IFS curves on SVM-order feature list. **c**: IFS curves on XGBoost feature list.
